# Expanding the Phenotypic Spectrum of *PAX6* Mutations: From Congenital Cataracts to Nystagmus

**DOI:** 10.3390/genes12050707

**Published:** 2021-05-09

**Authors:** Maria Nieves-Moreno, Susana Noval, Jesus Peralta, María Palomares-Bralo, Angela del Pozo, Sixto Garcia-Miñaur, Fernando Santos-Simarro, Elena Vallespin

**Affiliations:** 1 Department of Ophthalmology, Hospital Universitario La Paz, 28046 Madrid, Spain; susana.noval@salud.madrid.org (S.N.); jesusm.peralta@salud.madrid.org (J.P.); 2 Department of Molecular Developmental Disorders, Medical and Molecular Genetics Institue (INGEMM) IdiPaz, CIBERER, Hospital Universitario La Paz, 28046 Madrid, Spain; maria.palomares@salud.madrid.org; 3 Department of Bioinformatics, Medical and Molecular Genetics Institue (INGEMM) IdiPaz, CIBERER, Hospital Universitario La Paz, 28046 Madrid, Spain; angela.pozo@salud.madrid.org; 4 Department of Clinical Genetics, Medical and Molecular Genetics Institue (INGEMM) IdiPaz, CIBERER, Hospital Universitario La Paz, 28046 Madrid, Spain; sixto.garcia@salud.madrid.org (S.G.-M.); fernando.santos@salud.madrid.org (F.S.-S.); 5 Department of Molecular Ophthalmology, Medical and Molecular Genetics Institue (INGEMM) IdiPaz, CIBERER, Hospital Universitario La Paz, 28046 Madrid, Spain; elena.vallespin@salud.madrid.org

**Keywords:** *PAX6*, aniridia, congenital cataracts, nystagmus

## Abstract

Background: Congenital aniridia is a complex ocular disorder, usually associated with severe visual impairment, generally caused by mutations on the *PAX6* gene. The clinical phenotype of *PAX6* mutations is highly variable, making the genotype–phenotype correlations difficult to establish. Methods: we describe the phenotype of eight patients from seven unrelated families with confirmed mutations in *PAX6*, and very different clinical manifestations. Results: Only two patients had the classical aniridia phenotype while the other two presented with aniridia-related manifestations, such as aniridia-related keratopathy or partial aniridia. Congenital cataracts were the main manifestation in three of the patients in this series. All the patients had nystagmus and low visual acuity. Conclusions: The diagnosis of mild forms of aniridia is challenging, but these patients have a potentially blinding hereditary disease that might present with a more severe phenotype in future generations. Clinicians should be aware of the mild aniridia phenotype and request genetic testing to perform an accurate diagnosis.

## 1. Introduction

Congenital aniridia is a rare genetic disease that affects up to 1 in 64,000 people worldwide [1]. It is a panocular disorder characterized typically by iris and foveal hypoplasia, resulting in nystagmus and reduced visual acuity. Aniridia usually presents in early infancy and might be associated with other congenital anomalies such as congenital cataracts, and later-onset ocular abnormalities like glaucoma, aniridia-related keratopathy and cataracts. When the classical aniridia phenotype is present, the diagnosis is straight forward and most commonly caused by heterozygous mutations on the *PAX6* gene.

*PAX6*, paired box gene 6 (MIM#607108), is a member of the paired box gene family, which encodes a transcriptional regulator involved in the development of eye and central nervous tissues. *PAX6* is required for the formation of the lens placode, an ectodermal thickening that precedes lens development. The *PAX6* gene encodes a transcriptional regulator that recognizes target genes through its paired-type DNA-binding domain. The paired domain is composed of two distinct DNA-binding subdomains, the N-terminal subdomain, and the C-terminal subdomain, which bind respective consensus DNA sequences.

The PAX6 transcription factor is essential for the development of the visual system, the CNS, olfactory bulb, pituitary and pineal glands and pancreatic endocrine function [2,3,4]. The loss of *PAX6* function leads to the eyeless phenotype in *Drosophila* [5]. Haploinsufficiency of *PAX6* in mice causes the congenital condition aniridia, with defects in each of these organs and systems. Differentiation of the lens, cornea, iris and ciliary body are specifically affected by *PAX6* levels [6,7]. The mouse mutation *Small eye* (*Sey*), which has been proposed as a model for aniridia, results from defects in *PAX6*, and the human aniridia and murine Small eye phenotypes arise from homologous defects in *PAX6* [8]. *PAX6* is expressed not only in the optic field and in the lens but also in several brain regions and in the pancreas. *PAX6* mutations cause, in addition to ocular diseases, behavioral and neurodevelopmental phenotypes as well as disorders of the pancreas [9,10].

However, the clinical phenotype in aniridia is highly variable even between patients within the same family. Over the last few years, a mild or atypical aniridia phenotype has been described; in these cases the entire iris may be normal or have subtle abnormalities such as corectopia or ectropion uvea, challenging the diagnosis that can be delayed or overlooked [11].

The different *PAX6* mutations associated phenotypes had been classified in the literature as follows [12]:

(a) Classical aniridia: Characterized by partial or complete aniridia, nystagmus and foveal hypoplasia and later-onset cataracts, aniridia-related keratopathy and glaucoma. This is usually caused by premature termination codon mutations (nosense, frameshift and splicing);
(b) WARG (Willms tumour, anirida, genitourinary abnormalities and mental retardation). This is caused by large 11p deletions including *PAX6* and *WT1* or chromosomal rearrangements;
(c) Isolated foveal hypoplasia and nystagmus, usually caused by missense mutations;
(d) Microphthalmia, anophthalmia and coloboma;
(e) Anterior segment dysgenesis.

Patients with mutations affecting *PAX6* can present with a wide range of ocular features affecting both the anterior and posterior segment, making the genotype–phenotype correlations difficult to establish. In this case series, we report eight cases from seven unrelated families of patients with confirmed mutations affecting *PAX6*, and very different phenotypes.

## 2. Material and Methods

### 2.1. Patient Recruitment and Clinical Evaluation

The medical records of patients with an identified mutation in *PAX6* gene were included in this retrospective case series. The purpose was to describe the different phenotypes in patients with a confirmed *PAX6* mutation. The study was conducted following the principles of the Helsinki Declaration and was approved by the hospital’s ethics committee (C-GEN-007). All patients signed a consent form prior to participation in the study.

Clinical data from patients’ medical records were collected, including best-corrected visual acuity, anterior segment examination, intraocular pressure, and fundoscopy examination. When available, macular optical coherence tomography (OCT) and anterior segment or posterior segment photography were included.

### 2.2. Genetic Testing

Genomic DNA was isolated from peripheral blood in the preanilitic area in our institute with commercial Chemagic MSM I (Chemagen, PerkinElmer, Baesweiler, Germany). DNA quantity was assessed by spectrofluorometer quantification using TECAN M200 Infinite Pro Microplate Reader.

The DNA was subjected to a mutation test using a customized next-generation sequencing (NGS) gene panel containing 341 genes associated with eye pathology of suspected genetic origin, including *PAX6*. This panel was designed with NimbleDesign software (https://design.nimblegen.com, Roche NimbleGen, Inc. Pleasanton, CA, USA, accessed on 1 February 2021). The target bases covered 95.5% and the size was 1,003,106 Kb. For each sample, 150 bp paired-end libraries were created according to the standard protocols of NGS KAPA HTP Library Kit for Illumina^®^ platforms, SeqCap EZ Library SR (Roche NimbleGen, Inc. USA) and NEXTflex-96 Pre Capture Kit (Bioo Scientific, Phoenix, AZ, USA) for indexing. DNA from the captured sample was sequenced on a NextSeq 500 instrument (Illumina, San Diego, CA, USA) according to standard operating protocol. Validation of the variants was performed by Sanger sequencing.

For the analysis of the results, the INGEMM clinical bioinformatics team designed a bioinformatics analysis system to identify point polymorphisms (SNV, single nucleotide variant), insertions and deletions of small DNA fragments, as well as larger structural variants in the capture regions included in the next-generation sequencing panels. The system comprises a sample pre-processing step, alignment of reads to a reference genome, identification and functional annotation of variants, and variant filtering. All these steps use open tools widely used in the scientific community as well as proprietary tools. Furthermore, all phases are designed in a robust way including statistical parameters that inform about the status of the process and the convenience of continuing with the analysis. This allows the monitoring of the process and the appropriate quality controls to issue a reliable report on the aforementioned variants. Finally, the system backs up the raw and processed data. This data is stored in a database with encrypted and anonymized records to preserve patient confidentiality.

Software and versions used are as follows: Trimmomatic-0.32, Bowtie2-align version 2.1.0, Picard-tools 1.106, SAMtools version.0.1.19-44428cd, BEDtools v2.18.1, GenomeAnalysisTK version 3.3-0. Databases used are as follows: dbNSFP version 3.0, dbSNP v138, ClinVar date 20140703, SnpE 4.1l, Exac r0.3, SIFT ensembl 66, Polyphen-2 v2.2.2, MutationAssessor, release 2, FATHMM, v2.3, CADD, v1.3 and dbscSNV1.1. For the classification of the variants, the ACMG (American College of Medical Genetics and Genomics) criteria have been followed [13].

Array experiments were performed with a custom made array based on Agilent Technologies, called KaryoArray^®^. We selected 60-mer oligonucleotide features from Agilent’s eArray (Agilent, Santa Clara, CA, USA, https://earray.chem.agilent.com/earray, accessed on 15 December 2019) probe library in a custom high resolution format of 8 × 60 K. The array comprised specific probes covering all microdeletion and duplication syndromes, telomeres and peri-centromeric regions and also probes of backbone. The average density of the probe coverage is 43 kb. This focused oligonucleotide chip covers more than 350 clinically relevant regions of genomic imbalance.

In the design we consulted the Database of Genomic Variants (http://projects.tcag.ca/variation/, accessed on 1 February 2021) and DECIPHER (http://decipher.sanger.ac.uk/, accessed on 1 February 2021) to avoid CNVs with no apparent clinical relevance.

Array experiments were performed as recommended by the manufacturer (Agilent Technologies, Santa Clara, CA, USA). DNAs (500 ng) from the specimen and a reference of the same sex (Promega, Madison, WI, USA) were double-digested with RsaI and AluI for 2 h at 37 °C. After heat inactivation of the enzymes at 65 °C for 20 min, each digested sample was labeled by random priming (Genomic DNA Enzymatic Labeling Kit Agilent catalogue # 5190-0449) for 2 h using Cy5-dUTP for patient DNAs and Cy3-dUTP for reference DNAs. Labeled products were column purified (Microcon Ym-30 filters, Millipore Corporation, Burlington, MA, USA). After probe denaturation and pre-annealing with Cot-1 DNA, hybridization was performed at 65 °C with rotation for 24 h. After two washing steps, the microarray was then scanned by the Agilent Microarray Scanner and analyzed with Feature Extraction Software (v9.1 Agilent Technologies) and Genomic Work Bench 5.0.

The analysis and visualization of Karyoarray^®^ data was performed using Agilent Genomic WorkBench 5.0. A comprehensive description of the statistical algorithms is available in the user’s manual provided by Agilent Technologies. The aberration detection method 2 (ADM-2) quality weighted interval score algorithm identifies aberrant intervals in samples that have consistently high or low log ratios based on their statistical score. The score represents the deviation of the weighted average of the normalized log ratios from its expected value of zero calculated with derivative log2 ratio standard deviation algorithm. A fuzzy zero algorithm is applied to incorporate quality information about each probe measurement. Our threshold settings for the CGH analytics software to make a positive call were 6.0 for sensitivity, 0.35 for minimum absolute average log ratio per region, and 3 consecutive probes with the same polarity were required for the minimum number of probes per region.

## 3. Results

Eight patients of seven unrelated families were included in this study. Only two patients presented the full classical description of *PAX6*-related aniridia: aniridia, nystagmus, and foveal hypoplasia, while the other two presented manifestations presented in the aniridia spectrum: partial aniridia or aniridia-related keratopathy. One of the patients with classical aniridia ocular phenotype was diagnosed with WARG. All the *PAX6* variants including a deletion were found in a heterozygous state. The phenotype and genotype of each patient is summarized in Table 1.

### 3.1. Case 1

Ocular phenotype: aniridia, congenital cataracts, nystagmus, foveal hypoplasia, high myopia.

A 7-month-old boy presented with bilateral congenital cataract, aniridia, and nystagmus. He had no relevant family history and his parents were not consanguineous. He was able to fix and follow a small toy and he had horizontal nystagmus with left gaze torticollis (gaze towards the nystagmus’ nulls point). On anterior segment examination, he had bilateral anterior polar cataracts with rests of persistent pupillary membrane and complete aniridia. The fundus exam revealed bilateral foveal hypoplasia. 

At 3 years old he had developed high myopia with a cycloplegic refraction of −10 diopters in both eyes, and his visual acuity was 0.1 on each eye using contact lenses. The examination remained otherwise unchanged and there were no signs of keratopathy or limbal insufficiency.

Mutation: *PAX6*: NM_001258462.3:c.1183+1G>A (chr11:31812257, hg19), heterozygous. ACMG criteria: pathogenic. Inheritance: de novo. This variant has been described as pathogenic by one submitter in ClinVar, accession: VCV000492996.1, and variation ID:492996 in four patients with aniridia 1.

### 3.2. Case 2

Ocular phenotype: partial aniridia, nystagmus, posterior embryotoxon, foveal hypoplasia.

A two-month-old boy was referred to our clinic for bilaterally dilated pupils. He had a family history of congenital nystagmus in his mother, his mother’s sisters, and his grandfather. He had a low amplitude high-frequency nystagmus. On examination, he had bilateral partial aniridia and posterior embryotoxon. The fundus examination revealed bilateral foveal hypoplasia. His intraocular pressure was normal. His cycloplegic refraction was −1 diopters in both eyes.

Mutation: *PAX6*:NM_001258462.3:c.395C>G:p.(Pro132Arg) (chr11:31823113, hg19) heterozygous. ACMG criteria: likely pathogenic. Inheritance: maternal. This variant has been described as pathogenic by Sonoda et al [14] in a family with congenital nystagmus associated with a variant form of aniridia.

### 3.3. Case 3

Ocular phenotype: aniridia, nystagmus, anterior piramidal cataracts, optic nerve hypoplasia, foveal hypoplasia, aniridia-related keratopathy, high myopia.

Systemic phenotype: aortic coarctation.

A 6 week-old-boy was referred to our clinic for bilateral aniridia and cataracts with a confirmed genetic diagnose of WARG syndrome. He had no relevant family history. At birth he was diagnosed with aortic coarctation and had surgical treatment at four days of age, the genetic/environmental cause of the aortic coarctation had not been determined. On anterior segment examination, he presented bilateral aniridia, limbal insufficiency and piramidal cataracts, the funduscopy revealed bilateral optic nerve and foveal hypoplasia. An abdominal ultrasound was performed and he had a renal cyst in each kidney, and Willms tumor was ruled out. On his follow up, at nine months old, he was able to fix and follow a small object, he presented nystagmus and his cycloplegic refraction was −6 dp in his right eye and −10 dp in his left eye, the rest of the examination remained unchanged.

KaryoArray^®^ identified a copy-number loss spanning 13.48 Mb 11p14.1p12(28464346_41944418), NCBI Build 37. Also, array CGH analysis was performed on both parents and neither parent was found to carry the deletion.

The deletion identified in this case encompasses the following genes: *KCNA4, FSHB, C11orf46, MPPED2, DCDC1, DNAJC24, IMMP1L, ELP4, PAX6, RCN1, WT1, WIT1, EIF3M, CCDC73, PRRG4, QSER1, DEPDC7, TCP11L1, LOC283267, CSTF3, HIPK3, C11orf41, C11orf91, CD59, FBXO3, LMO2, CAPRIN1, NAT10, ABTB2, CAT, ELF5, EHF, APIP, PDHX, CD44, SLC1A2, PAMR1, FJX1, TRIM44, LDLRAD3, COMMD9, PRR5L, TRAF6, RAG1, RAG2, C11orf74, and LRRC4C.*

### 3.4. Case 4

Ocular phenotype: nystagmus, foveal hypoplasia, aniridia-related keratopathy.

A 29-year-old woman, related to case 2 (aunt), followed in our clinic for nystagmus, foveal hypoplasia, and limbal insufficiency. Her visual acuity was 0.16 on her right eye and 0.1 on her left eye. On examination, she had severe limbal insufficiency in both eyes with peripheral and central aniridia-related keratopathy grade 3 [16]. The iris morphology was normal in both eyes. The intraocular pressure was normal and on the fundus exam, she had bilateral foveal hypoplasia.

Mutation: *PAX6*:NM_001258462.3):c.395C>G:p.(Pro132Arg) (chr11:31823113, hg19) heterozygous. ACMG criteria: likely pathogenic. Inheritance: paternal. This variant has been described as pathogenic by Sonoda et al [14] in a family with congenital nystagmus associated with a variant form of aniridia.

### 3.5. Case 5

Ocular phenotype: congenital cataracts, nystagmus, secondary glaucoma.

A two-week-old boy presented with bilateral congenital cataracts. On examination, he had bilateral dense anterior cataracts that hindered the retinal examination, with no iris anomalies. The posterior pole was normal on the ocular ultrasound. He underwent cataract surgery with no IOL insertion at 6 weeks on his right eye and 7 weeks on his left eye. A few weeks later he developed secondary glaucoma on both eyes, he had a bilateral trabeculectomy first and Ahmed drainage implant on his right eye 8 months later.

At five years old his best-corrected visual acuity is 0.1 on each eye. On examination, he had sensory nystagmus with left gaze torticollis. His fundus examination was normal with no foveal hypoplasia. He had a cup/disc ratio of 0.2 on his right eye and 0.1 on his left eye, and the intraocular pressure was 16 mmHg in both eyes, using timolol 0.5% twice a day on his right eye.

Mutation: *PAX6*:NM_001258462.3:c.77G>A:p.(Arg26Gln) (chr11:31824316, hg19), heterozygous. ACMG criteria: likely pathogenic. Inheritance: de novo. This variant has been described as pathogenic by Williamson et al [15] in a family with bilateral microphthalmia, bilateral iris coloboma, and bilateral congenital cataracts. One of the patients in this family also had nystagmus and secondary glaucoma.

### 3.6. Case 6

Ocular phenotype: nystagmus, chorioretinal coloboma, foveal hypoplasia, high myopia, rod retinal dystrophy.

A six-month-old boy came to our clinic for a second opinion. He was born at 39 weeks from a spontaneous pregnancy of non-consanguineous parents. He had a history of nystagmus, which was first noted by his parents at 2 months old. On the first examination, he was able to fix and follow a small toy with both eyes, his pupils were hyporeactive and he had jerk nystagmus with a vertical component. The anterior segment was normal with no iris or lens abnormalities. On fundoscopy, he had a small chorioretinal coloboma inferior to the optic disc in his left eye and foveal hypoplasia in both eyes. The cycloplegic refraction showed low myopia. The neurological examination was normal and the visual evoked potential showing a low amplitude in both eyes.

At six years old his vision was 0.1 (decimal) in both eyes and he had developed −10 sphere diopters, and the fundus revealed a hypo-pigmented retina with bilateral foveal hypoplasia. Retinal dystrophy was suspected and the electroretinogram showed low amplitude in the rods responses, with normal oscillatory potentials and cones responses.

Mutation: *PAX6*:NM_001258462.3:c.262A>G:exon7:p.(Ser88Gly) (chr11:31823246, hg19), heterozygous. ACMG criteria: likely pathogenic. Inheritance: de novo. This variant has not been previously described in the literature.

### 3.7. Case 7

Ocular phenotype: microphthalmia, congenital cataracts, nystagmus.

Systemic phenotype: microcephaly.

A two-and-a-half-month-old girl was referred to our clinic for bilateral congenital cataracts. On examination, her visual acuity was at least light perception and she had low amplitude nystagmus. On the anterior segment exam, she had bilateral dense anterior cataracts that hindered the retinal examination. Her iris configuration was normal. An ultrasound revealed a normal posterior pole with an axial length of 16 mm in both eyes. She had a bilateral lensectomy without intraocular lens (IOL) insertion at 3 months old. She was referred to the pediatrician who noted microcephaly with a normal brain and abdominal ultrasound. The fundus examination after bilateral cataract extraction was normal with no signs of foveal or optic nerve hypoplasia.

Mutation: *PAX6*:NM_001258462.3:c.219G>T:(p.Arg73Ser) (chr11:31823289, hg 19), heterozygous. ACMG criteria: likely pathogenic. Inheritance: no parents available. This variant has not been previously described in the literature.

### 3.8. Case 8

Ocular phenotype: nystagmus, corectopia, ectropion uvea, congenital cataracts, foveal hypoplasia. 

Systemic phenotype: bronchiectasis, type-II diabetes, encephalopathy, epilepsy, Perthes disease.

A 14-year-old girl presented with nystagmus and congenital cataracts. She had a family history of congenital cataract and nystagmus in her father and sister, and her sister also has an iris coloboma. She had a medical history of bronchiectasis, type-II diabetes, encephalopathy, epilepsy, and Perthes disease, no other genetic diagnosis has been associated to her systemic phenotype, but she continues to be under investigation. Her visual acuity was 0.1 in her right eye and 0.05 in her left eye. She had bilateral microphthalmia and horizontal nystagmus, right gaze torticollis (gaze towards the nystagmus’ nulls point), and 30 diopters exotropia. The anterior segment examination showed a bilateral corectopia with ectropion uvea and anterior polar cataracts. On fundoscopy, she had foveal hypoplasia and peripheral vascular thinning. The cycloplegic refraction was −14 sphere diopters in both eyes.

Mutation: *PAX6*:NM_001258462.3:c.398G>T:p.(Ser133Ile) (chr11:31823110, hg19), heterozygous. ACMG criteria: likely pathogenic. Inheritance: paternal. This variant has not been previously described in the literature.

## 4. Discussion

The function of the PAX6 protein is crucial in human eye development, and patients affected with *PAX6* mutations showed a wide variety of congenital ocular disorders, often resulting in nystagmus and severe visual impairment. In our case series, seven out of eight patients had nystagmus, and visual acuity was reduced in all the patients where visual acuity testing was possible to measure due to collaboration.

Three new likely pathogenic mutations have been detected in the *PAX6* gene.

The variations p.Ser88Gly and p.Ser133Ile have not been reported in the literature in individuals with PAX6-related disease. The p.Ser88Gly change replaces serine with cysteine at codon 88 of the PAX6 protein, and the p.Ser133Ile change replaces serine with isoleucine at codon 133 of the PAX6 protein. These variants are not present in population databases (ExAC and GnomAD no frequency). Algorithms developed to predict the effect of missense changes on protein structure and function (SIFT, PolyPhen-2, Align-GVGD) all suggest that these variants are likely to be disruptive, but these predictions have not been confirmed by published functional studies and their clinical significance is uncertain. In summary, thanks to the description of our patients and the confirmation that the variant p.Ser88Gly is de novo, and p.Ser133Ile is inherited from the father who has congenital cataract and nystagmus, we consider that both variants are likely pathogenic.

In the mutation p.Ser380ArgfsTer20, the sequence change creates a premature translational stop signal in the *PAX6* gene. The deletion causes a frameshift starting with the codon serine 380, changes this amino acid to an arginine residue and creates a premature stop codon at position 20 of the new reading frame, denoted p.Ser380ArgfsTer20. This likely pathogenic variant is predicted to cause loss of normal protein function either through protein truncation or nonsense-mediated mRNA decay. It is expected to result in an absent or disrupted protein product. This variant is not present in population databases (ExAC and GnomAD no frequency) and is inherited de novo in our patient. The loss-of-function variants in *PAX6* are known to be pathogenic. For these reasons, this variant has been classified as likely pathogenic.

Only four cases in this study (1, 2, 3, and 4) presented with the classical aniridia phenotype, aniridia or aniridia-related keratopathy, nystagmus, and foveal hypoplasia. Classical aniridia is usually associated with deletions or premature protein truncations, while other aniridia phenotypes are associated with missense changes.

The sporadic cases of aniridia might have an increased risk of developing a nephroblastoma or Wilms tumor (WARG syndrome). This syndrome is caused by large chromosomal deletions in chromosome 11p13, including *PAX6* and the Wilms tumor gene (*WT1*), this genotype represents 10% of all classical aniridia cases [12]. In patients with aniridia secondary to mutations only in *PAX6*, investigations for Wilms tumor are not necessary [17]. In young infants with the classical aniridia phenotype we recommend performing an abdominal ultrasound and a complete pediatric examination to rule out a nephroblastoma and genitourinary malformations before receiving the genetics results.

Five patients of our series had bilateral congenital cataracts, and it was the main ocular finding at diagnosis in three of those patients. This phenotype has not been classically associated with *PAX6* mutations. Bilateral congenital cataract usually has a good visual prognosis if surgery is performed between 6 and 8 weeks old and no other ocular abnormalities are seen [18]. Patients with *PAX6* mutations often had posterior pole abnormalities such as foveal hypoplasia, which might be difficult to diagnose in a young infant presenting with congenital cataracts, as this patient will characteristically present nystagmus at a low visual acuity, like case 4, 7 and 8. We believe that this phenotype should be recognized as a new entity within the *PAX6* classification, as *PAX6*-related congenital cataracts. Clinicians should be aware that mutations in the *PAX6* gene can initially present as bilateral congenital cataracts and search for the mutation, especially if the patient has low vision, nystagmus, and foveal hypoplasia.

Nystagmus was the most consistent ocular finding between the patients in this study. Even though many forms of hereditary infantile nystagmus have no sensory visual defect detectable, like X-linked forms of the *FRMD7* gene [19], a high proportion of patients initially classified as infantile nystagmus have underlying anatomical abnormalities affecting the eye or the optic nerve on close examination, on visual evoked potentials or on electroretinography. Vision loss in early infancy (before 2 years of age) is usually associated with nystagmus, the clinician should be aware and look carefully for abnormalities in every appointment, especially in infants and toddlers since at that age visual acuity is usually less reliable. All children with nystagmus should have a complete ophthalmological examination in every appointment including a slit lamp exam and fundoscopy. The use of optical coherence tomography (OCT) and electrophysiology testing in these patients with nystagmus and vision loss might reveal subtle forms of optic atrophy, albinism, congenital retinal dystrophies [20], or aniridia as described in this article.

The most frequent refractive error found in our patients was myopia or high myopia [21]. This finding, also described in other inherited eye diseases that cause low vision since a young age, is probably secondary to the emmetropization process being impaired since early childhood. A recently published study suggested that in patients with high myopia, *PAX6* mutations do not increase the risk of developing myopic maculopathy [22].

The clinical diagnosis of mild aniridia is challenging. In these cases, the iris is usually normal or nearly normal, with a subtle transillumination defect, partial defects, corectopia, ectropion uvea, or embryotoxon. This mild phenotype had been associated with less severe visual implications compared to classical aniridia, but these patients have a severe, potentially blinding, hereditary disease that might present with a more severe phenotype in future generations [1,11]. In the patients described in this series, even the mild phenotypes had severe visual impairment. Clinicians should be aware of this mild aniridia phenotype and the *PAX6*-related congenital cataracts and request genetic testing when suspected.

In summary, this manuscript describes the wide phenotypic spectrum of *PAX6* mutations, including a newly described phenotype, the *PAX6*-related congenital cataracts with no iris abnormalities present, and we have also described three new likely pathogenic mutations in the *PAX6* gene.

## Figures and Tables

**Table 1:** Phenotype and genotype characteristics of the patients.

		Case 1	Case 2	Case 3	Case 4	Case 5	Case 6	Case 7	Case 8
Genotype	Nucleotide Change	c.1183+1G>A	c.395C>G	arr[GRCh37] 11p14.1p12(28464346-41944418)x1	c.395C>G	c.77G>A	c.262A>G	c.219G>T	c.398G>T
	Protein Change	--	p.(Pro132Arg)	--	p.(Pro132Arg)	p.(Arg26Gln)	p.(Ser88Gly)	p.(Arg73Ser)	p.(Ser133Ile)
	Described by	ClinVar ID:492996	Sonoda et al [14]	ND ^2^	Sonoda et al [14]	Williamson et al [15]	ND ^2^	ND ^2^	ND ^2^
	Inheritance	De Novo	Maternal	De Novo	Paternal	De Novo	De Novo	N/A	Paternal
	Type of mutation	Splicing	Missense	Deletion	Missense	Missense	Missense	Missense	Missense
Ocular Phenotype	BCVA on follow-up (decimal)	0.1	N/A ^1^	Fix and Follow	0.16	0.1	0.1	0.1	0.1
	Nystagmus	Yes	Yes	Yes	Yes	Yes	Yes	Yes	Yes
	Anterior segment	Aniridia Congenital cataract	Partial aniridia Posterior embryotoxon	Aniridia Congenital cataract Aniridia related keratopathy	Aniridia related keratopathy	Congenital cataract Secondary glaucoma	Normal	Microphthalmia Congenital cataracts	Corectopia Ectropion uvea Congenital cataracts
	Posterior segment	Foveal hypoplasia	Foveal hypoplasia	Foveal hypoplasia Optic nerve hypoplasia	Foveal hypoplasia	Normal	Chorioretinal coloboma Foveal Hypoplasia Rod retinal dystrophy	Normal	Foveal hypoplasia
	Refractive error	High myopia	Myopia	High myopia	--	--	High myopia	--	High myopia
Systemic Phenotype		--	--	Aortic Coarctation Bilateral Renal cyst	--	--	--	Microcephaly	Bronchiectasis Type-II diabetes Encephalopathy Epilepsy Perthes disease

^1^ Not Available. ^2^ ND: Not described. Eight patients have been analyzed, of which one has a splicing mutation already described in the literature, three others have missense mutations already described in the literature and one patient with an atypical deletion of 13.48Mb and three missense variants is described for the first time. All changes are in heterozygosity.

## Data Availability

All data obtained are presented in this publication. No further data are available.
